# Plant Fungal Diseases and Crop Protection

**DOI:** 10.3390/jof11040274

**Published:** 2025-04-01

**Authors:** Ofir Degani

**Affiliations:** 1 Plant Sciences Department, MIGAL—Galilee Research Institute, Tarshish 2, Kiryat Shmona 1101600, Israel; d-ofir@migal.org.il or ofird@telhai.ac.il; Tel.: +972-54-678-0114; 2 Faculty of Sciences, Tel-Hai College, Upper Galilee, Tel-Hai 1220800, Israel

Fungi represent the largest group of plant pathogens, infecting their hosts via leaves, seeds, and roots. These pathogens cause significant crop damage through diverse mechanisms, leading to reduced global production and an ongoing demand for innovative control strategies [1,2]. Effective management of fungal diseases in crops depends upon a comprehensive understanding of the fungi involved, identifying susceptible growth stages, and recognizing environmental factors that influence disease progression [3].

Chemical pesticides have long been fundamental in crop protection strategies against phytopathogens [4]. Nevertheless, growing public concerns regarding synthetic chemical usage [5] and the emergence of fungicide-resistant fungal strains [6] necessitate the exploration of alternative, environmentally friendly approaches [7,8]. Despite these concerns, traditional chemical methods have proven highly effective, especially in severe disease outbreaks, benefiting from well-established production and supply chains, and are familiar to farmers and agricultural field guides. Therefore, integrating biological and chemical approaches (mixed-method management) has been proposed as a transitional strategy from conventional chemical interventions toward greener technologies [9,10,11]. Such combinations may also enhance the robustness and stability of protective microorganisms under varying environmental conditions, ultimately benefiting agricultural practices.

At present, there is an increasing global scientific emphasis on developing sustainable crop production methods capable of meeting growing demands without environmental harm. Utilizing resistant cultivars is a promising method due to its low cost and compatibility with various agricultural practices [12]. However, phytopathologists have noted the emergence of highly aggressive pathogen strains capable of overcoming cultivar resistance, thus compromising their effectiveness [13,14].

Recently, biological control agents (BCAs) have gained considerable attention as potential tools for managing fungal diseases [15,16,17]. Specific BCAs, such as *Trichoderma* spp., *Bacillus* spp., *Pseudomonas fluorescens*, *Beauveria bassiana*, and *Gliocladium* spp., offer numerous benefits for crop protection. Some BCAs function as beneficial endophytes, living symbiotically within plants [18,19,20].

Exploring innovative green technologies to enhance fungal pathogen control is a critical priority, and the full potential of these advancements is only beginning to unfold [21]. Cutting-edge technologies, including microbiome engineering, nanotechnology, artificial intelligence (AI), genome editing, RNA interference, and functional peptides, could facilitate precise and sustainable management strategies for fungal diseases in agriculture [22,23,24]. Microbiome-based solutions and nanoparticles enable targeted pathogen suppression with minimal environmental impacts, while AI and machine learning significantly enhance disease detection accuracy—such as analyzing remote-sensing data for field monitoring and treatment optimization [25,26]. Furthermore, advancements in molecular diagnostics, such as endpoint PCR and real-time PCR, have revolutionized accurate pathogen identification, enabling early detection and improved disease management strategies [27,28].

This Special Issue comprises two review articles and eleven original research papers, highlighting recent innovations and scientific advances in plant fungal diseases and their agricultural implications. It also includes discussions of non-pathogenic fungi associated with plants that influence disease onset and severity. The broad range of topics covered underscores the complex challenges we face in modern agriculture, including significant diseases affecting various hosts—from fruit trees and woody oil plants to staple crops, such as soybean, potato, wheat, strawberries, and even edible mushrooms, such as morels (*Morchella* spp.) and white button mushrooms (*Agaricus bisporus*). Furthermore, it explores innovative biocontrol methods and novel weed management approaches by mycoherbicide.

I extend my sincere appreciation to all contributing authors and reviewers whose valuable efforts were crucial for the successful publication of this Special Issue. Given the complexity and dynamic nature of ensuring global crop health, food security, and sustainable agriculture under global changes, including climate change and industrialization, there are significant challenges ahead in developing and optimizing effective fungal control technologies. The promising outcomes of ongoing research should encourage stakeholders and industry partners to actively translate these innovative management strategies into practical agricultural applications.
